# Data Resource Profile: Children in Wales: The Cohort for Health and Wellbeing Inequality (CWTCH)

**DOI:** 10.1093/ije/dyag180

**Published:** 2026-09-10

**Authors:** Sarah J Aldridge, Lateef Akanni, Stuart Bedston, Davara Bennett, Michelle Black, Yanhua Chen, Yu Wei Chua, Oluwaseun B Esan, Lucy J Griffiths, Emily Lowthian, Philip McHale, Ashley Akbari, David Taylor-Robinson

**Affiliations:** Population Data Science, Swansea University Medical School, Faculty of Medicine, Health & Life Science, Swansea University, Swansea, United Kingdom; Department for Public Health, Policy and Systems, University of Liverpool, Liverpool, United Kingdom; Population Data Science, Swansea University Medical School, Faculty of Medicine, Health & Life Science, Swansea University, Swansea, United Kingdom; Department for Public Health, Policy and Systems, University of Liverpool, Liverpool, United Kingdom; Department for Public Health, Policy and Systems, University of Liverpool, Liverpool, United Kingdom; Department for Public Health, Policy and Systems, University of Liverpool, Liverpool, United Kingdom; Department for Public Health, Policy and Systems, University of Liverpool, Liverpool, United Kingdom; Department for Public Health, Policy and Systems, University of Liverpool, Liverpool, United Kingdom; Population Data Science, Swansea University Medical School, Faculty of Medicine, Health & Life Science, Swansea University, Swansea, United Kingdom; Department of Education and Childhood Studies, School for Social Sciences, Swansea University, Swansea, United Kingdom; Department for Public Health, Policy and Systems, University of Liverpool, Liverpool, United Kingdom; Population Data Science, Swansea University Medical School, Faculty of Medicine, Health & Life Science, Swansea University, Swansea, United Kingdom; Department for Public Health, Policy and Systems, University of Liverpool, Liverpool, United Kingdom

Key Features

- Children in Wales: The Cohort for Health and Wellbeing Inequality (CWTCH) was developed to enable large-scale, longitudinal research into inequalities in child health and well-being. It supports research into the causes and consequences of these inequalities, including perinatal influences, and their interactions with education, social care, and physical and mental health from childhood into young adulthood.
- This data resource provides an e-cohort of 970 880 individuals born in Wales between 1 January 2000 and 31 December 2024, with links to mothers and households, alongside key demographic, perinatal, and health- and social-care-usage indicators.
- Hosted within the Secure Anonymised Information Linkage (SAIL) Databank trusted research environment, CWTCH can be linked to a wide range of administrative, health, education, and social-care data sources.
- A key feature of CWTCH is its integration of area-level deprivation indices (Welsh Index of Multiple Deprivation and Townsend) with individual- and household-level socio-economic measures, including eligibility for free school meals and, where available, linked census-derived indicators such as education, occupation, and housing tenure.
- The code used to create this e-cohort is publicly available on GitHub for accredited researchers wishing to recreate CWTCH within SAIL or adapt it for their own datasets.

## Data resource basics

### Background

Reducing health inequalities and improving child health are longstanding government priorities in Wales. Despite this, recent evidence suggests that existing strategies have not achieved a meaningful or sustained impact [1–3]. The scale of the challenge is stark: between 2022 and 2024, ∼31% of children in Wales were living in relative income poverty [4]. Socio-economic disadvantage is a fundamental determinant of child health and development, shaping outcomes across physical and mental health, education, and broader social well-being [5–10]. Children growing up in disadvantaged circumstances are disproportionately exposed to multiple, co-occurring risks, contributing to poorer health trajectories and increased risk of multimorbidity across the life course [10, 11].

Against a backdrop of persistently high child poverty across Wales and the wider UK, governments have renewed their focus on policy responses, including the introduction of a national child-poverty strategy [12]. However, significant challenges remain in translating policy ambition into measurable improvements. Critical evidence gaps include identifying sensitive periods for intervention; determining which populations should be prioritized; understanding which combinations of interventions are most effective and cost-efficient within constrained public finances; and establishing realistic time horizons over which benefits and cost savings emerge [13]. Addressing these questions requires robust, population-scale, longitudinal data capable of generating timely, policy-relevant evidence.

Routinely collected data from electronic health records, when linked across sectors, provide a powerful complement to traditional cohort studies. Unlike survey-based birth cohorts, which are often limited by sample size, attrition, and representativeness, linked administrative data can capture whole populations over extended periods. International initiatives demonstrate the potential of such approaches: the Danish DANLIFE cohort links population-wide health, education, and social records [14], while ECHILD (Education and Child Health Insights from Linked Data) links de-identified data from hospitals, schools, and social care in England [15]. These resources have already generated policy-relevant insights, such as identifying higher rates of preventable hospital admissions among children in deprived areas [16]. Within Wales, linked administrative data have similarly been used to examine child-protection trajectories and the relationship between adoption timing and educational outcomes [17].

Children in Wales: The Cohort for Health and Wellbeing Inequality (CWTCH) was established to address current limitations in the Welsh data landscape. It is constructed by using linked, routinely collected, anonymized population-scale data within the Secure Anonymised Information Linkage (SAIL) databank—a nationally trusted research environment (TRE) for Wales [18]. SAIL integrates health and administrative data, providing anonymized, linkable individual- and household-level data for Welsh general practice (GP)-registered residents [19], supporting the comprehensive, longitudinal analysis of health and social outcomes. SAIL represents ∼86% of the Welsh population, with 83% of GPs in Wales contributing data; this broad coverage is achieved through the default inclusion of new GP registrants, who are informed of their choice to opt out of sharing their anonymized data for research.

Previous Welsh child-focused data resources have made important contributions, but have key limitations. Born in Wales (2011 onwards) is restricted to children aged ≤11 years, hindering analyses of adolescent outcomes such as mental health, self-harm, substance use, and teenage pregnancy, as well as longer-term transitions into higher education and employment [20]. The Growing Up in Wales study examined gestational and postnatal environmental risk factors with rich data collection, but its small sample size restricts population-level analyses [21]. These cohorts, while valuable, have addressed specific research questions rather than providing comprehensive population-scale infrastructure. More broadly, while numerous studies have used SAIL data to investigate child health, these have typically been conducted without a consistent cohort framework, resulting in fragmentation, reduced comparability, and challenges in replication across studies [22].

CWTCH addresses these gaps by providing a unified, population-scale, longitudinal cohort infrastructure designed to support reproducible and policy-relevant research on child and maternal health inequalities in Wales. CWTCH was developed as part of a National Insitute for Health and Care Research (NIHR)funded research project led by the University of Liverpool, in partnership with Swansea University. The cohort is specifically designed to investigate pathways into health inequalities across childhood into early adulthood, in order to inform policy.

### Who is in CWTCH?

CWTCH is a population-scale, research-ready data asset (RRDA) comprising all children born in, or resident in, Wales before their eighteenth birthday, with a recorded week of birth of between 1 January 2000 and 31 December 2024. The cohort also includes linked maternal records and residential information, enabling analyses at the individual, maternal, and household levels. RRDAs are created through the systematic integration, cleaning, and harmonization of multiple raw data sources to create a consistent, analysis-ready infrastructure tailored to support specific research aims. Each child and mother is assigned a unique Anonymised Linkage Field (ALF) to facilitate linkage with other SAIL datasets throughout their follow-up. In addition to person-level linkage, Residential Anonymised Linkage Fields (RALFs) enable linking individuals to their households or places of residence [23]. Together, these linkage mechanisms support the longitudinal tracking of children, their mothers, and their living environments across multiple sectors, including healthcare, education, and social care. All data are accessed by accredited researchers via a TRE, ensuring high standards of data security and governance.

**Figure 1:**
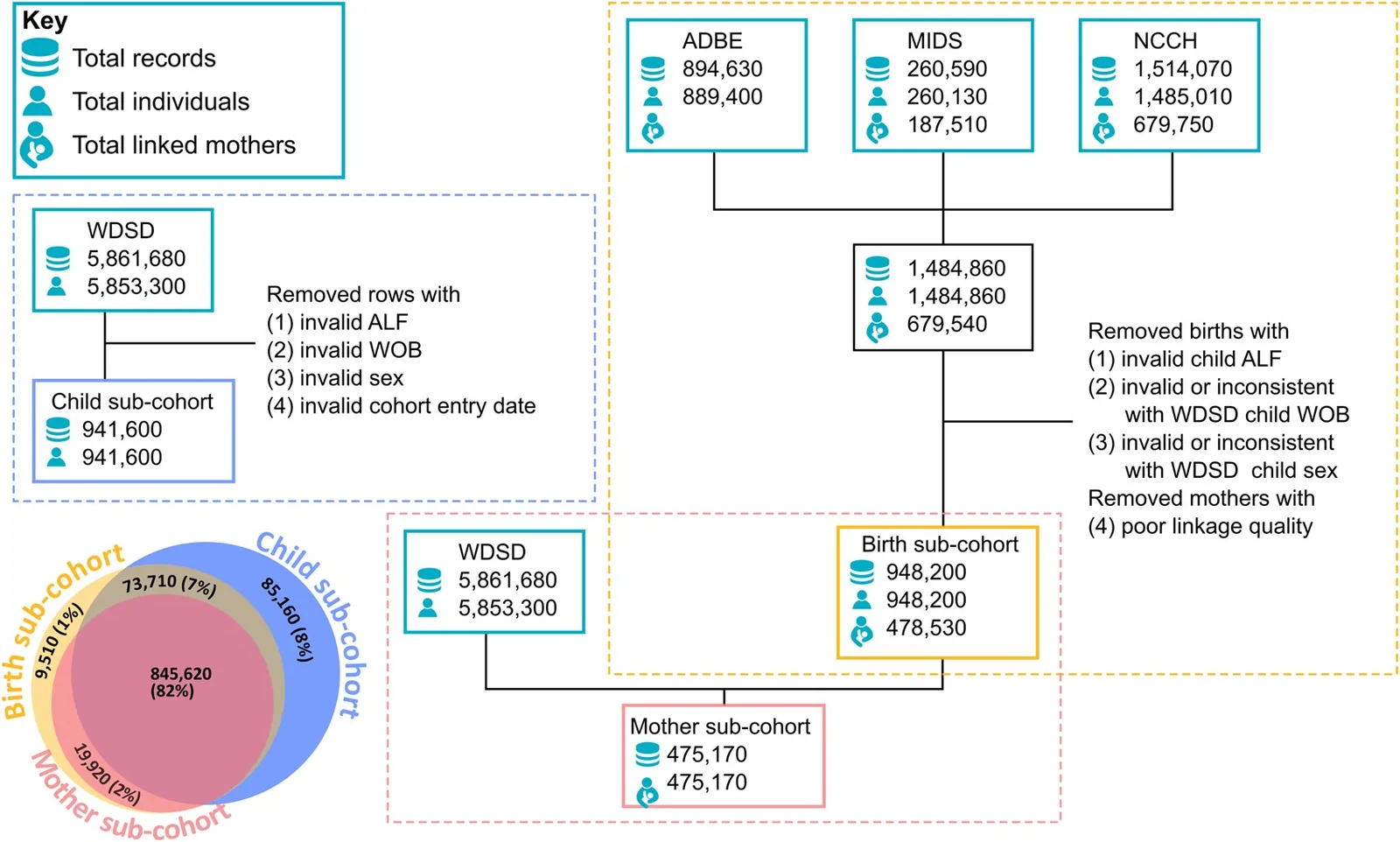
Consort diagram for the three CWTCH sub-cohorts. The Child sub-cohort is formed from all valid records in the Welsh Demographic Service Dataset (WDSD). The Birth sub-cohort is derived from all valid records in birth datasets: Annual District Birth Extract (ADBE), Maternity Indicators Dataset (MIDS), and National Community Child Health (NCCH). The Mother cohort is formed from all linked mothers in the Birth sub-cohort with a record in WDSD. ALF, Anonymised Linkage Field; WOB, week of birth.

CWTCH is structured by using three core, linkable sub-cohorts: a Birth sub-cohort, a Child sub-cohort, and a Mother sub-cohort. The Birth sub-cohort comprises individuals with a birth record in Wales, capturing fixed demographic and birth-relevant information (e.g. birthweight), and includes a linkage field to the Mother sub-cohort, which contains fixed maternal demographic information. The Child sub-cohort uses GP registration data to identify all children who have lived in Wales before their nineteenth birthday and is not limited to those with a birth record—thereby capturing children who moved to Wales as well as those with missing birth records. These sub-cohorts are not mutually exclusive; there is considerable overlap between the Child and Birth sub-cohorts, and between these and the Mother sub-cohort for mothers born after 2000. Together, these components form a flexible, population-wide e-cohort enabling longitudinal analysis spanning preconception, pregnancy, childhood, adolescence and into adulthood (Supplementary S1). The cohort includes 970 880 children (Child and Birth sub-cohorts combined), of whom 843 840 (87%) have both a birth and follow-up GP records. The Mother sub-cohort contains 475 170 mothers who link to 859 900 children (Fig. 1).

Membership of CWTCH is dynamic. Individuals enter the cohort either at birth [appearing in Annual District Birth Extract (ADBE), Maternity Indicators Dataset (MIDS), or National Community Child Health (NCCH)] or when they are registered with a SAIL GP in Wales [appearing in the Welsh Demographic Service Dataset (WDSD)], allowing additional children and mothers to join longitudinally. Data sources used to provide birth details are refreshed quarterly, death information is refreshed monthly, and the data source for GP registration is updated weekly (Supplementary S2). These update cycles enable full cohort refreshes on a quarterly basis, ensuring that participant inclusion, follow-up time, and outcome capture remain current and complete. The dynamic structure also allows individuals to move within Wales, exit the cohort when they emigrate or deregister from Welsh GP services, and re-enter if they subsequently re-register.

CWTCH provides extensive longitudinal follow-up. The Child and Birth sub-cohorts together contribute >19.6 million person-years of observation (median 10.1 years). Of the 970 880 children in CWTCH (Birth and Child sub-cohorts combined), 811 460 had complete follow-up from cohort entry to study end, with exits primarily due to moving out of Wales (129 250), loss to follow-up (28 480), or death (1690), and the exit rates were highest among children aged <4 years and those aged 18–19 years.

The Mother sub-cohort contributes 10.9 million person-years of follow-up from cohort entry to study end, with a median duration of 24 years (interquartile range (IQR): 24–24). Of the 475 010 mothers, 416 130 had complete follow-up, with cohort exits due to moving out of Wales (54 840) or death (4040). The median age at cohort entry was 17 years (IQR: 6–24) and that at exit was 41 years (IQR: 35–49).

Demographic characteristics of the sub-cohorts are summarized in Table 1. Among the 947 800 individuals in the Birth sub-cohort, 51% were male and 83% were recorded as White. Fewer than 1% had a death recorded before the end of follow-up. The Child sub-cohort included 940 800 individuals, with similar proportions for sex and ethnic group. Among mothers, 88% were White and 1% had died before the end of follow-up.

**Table 1:** Fixed characteristics across the three cohorts, including flags for whether they are registered with a SAIL GP, which SAIL data sources they appear in [primary care: Welsh Longitudinal General Practice Dataset (WLGP), secondary care: Patient Episode Dataset for Wales (PEDW), Children Receiving Care and Support Census (CRCS), Looked After Children Wales (LACW)], whether they are on the child-protection register, have ever received free school meals (FSM), and have ever required special educational needs (SEN). A dash indicates the data is not applicable.

Characteristic	Value	Birth sub-cohort [*n* (%)]	Child sub-cohort [*n* (%)]	Mother sub-cohort [*n* (%)]
**Total**	**Total**	**947 800**	**940 800**	**475 010**
Sex	Male	486 530 (51)	482 540 (51)	–
	Female	461 280 (49)	458 260 (49)	–
Ethnic group	White	789 550 (83)	779 060 (83)	416 780 (88)
	Mixed	26 500 (3)	26 470 (3)	5330 (1)
	Asian	38 550 (4)	40 160 (4)	18 730 (4)
	Black	14 700 (2)	15 300 (2)	6430 (1)
	Other	17 540 (2)	18 420 (2)	7520 (2)
	Unknown	60 980 (6)	61 400 (7)	20 230 (4)
Deaths	Total	3960 (<1)	1750 (<1)	4160 (1)
Recorded in WLGP	Total	918 560 (97)	940 300 (100)	474 990 (100)
Has a SAIL GP	Total	816 000 (86)	832 430 (88)	438 780 (92)
Recorded in PEDW	Total	583 980 (62)	578 130 (61)	447 860 (94)
Record in CRCS	Total	25 180 (3)	25 300 (3)	1040 (<1)
Recorded in LACW	Total	16 200 (2)	15 850 (2)	2520 (1)
Child-protection-register flag	Total	6600 (1)	6600 (1)	200 (0)
FSM flag	Total	224 280 (24)	225 430 (24)	34 420 (7)
SEN flag	Total	193 980 (20)	194 950 (21)	42 530 (9)

Birth-specific outcomes (Table 2) reveal that 99% of recorded births were live births, with 2% involving multiple gestations. Approximately 70% of births were within the normal weight range and 59% occurred at full term (37–42 weeks’ gestation). These distributions are broadly consistent with expected population patterns and support the representativeness of the cohort for perinatal analyses.

**Table 2:** Counts and proportions of each field for a selection of birth-specific characteristics in the Birth sub-cohort.

Characteristic	Value	Count	Proportion (%)
Total	Total	947 800	100
Birth category	Live birth	944 570	>99
	Still birth	3230	<1
Multiple gestation	Singleton	843 770	89
	Multiple gestation	21 630	2
	Unknown	82 410	9
Mother’s WIMD at baby’s birth	1—most deprived	197 760	21
	2	164 230	17
	3	167 650	18
	4	133 920	14
	5—least deprived	126 930	13
	Unknown	157 320	17
Gestational age	Extremely premature (EPT) (<28 weeks)	4520	<1
	Very premature (VPT) (28–31 weeks)	7620	1
	Premature (PT) (32–36 weeks)	52 270	6
	Term (T) (37–40 weeks)	558 280	59
	Late term (LT) (41 weeks)	151 640	16
	Very late term (VLT) (>42 weeks)	35 530	4
	Unknown	137 950	15
Birthweight	Extremely low birthweight (ELBW) (<1000 g)	6460	1
	Very low birthweight (VLBW) (1000–1499 g)	6180	1
	Low birthweight (LBW) (1500–2499 g)	50 690	5
	Normal weight (NW) (2500–3999 g)	667 980	70
	High birthweight (HBW) (4000–4499 g)	82 320	9
	Very high birthweight (VBHW) (≥4500 g)	13 990	1
	Unknown	120 190	13

WIMD, Welsh Index of Multiple Deprivation.

## Data collected

Addressing inequalities is a central motivation for the design and development of CWTCH, which draws on several data sources available within SAIL (Table 3). The RRDA integrates a wide range of harmonized indicators across health, education, and social domains, enabling the comprehensive investigation of inequalities at both area and individual levels (Supplementary S2).

**Table 3:** The data sources used to create CWTCH in SAIL, with their acronym and purpose.

Dataset	Abbreviation	Data
**Annual District Death Extract**	ADBE	Register of all deaths relating to Welsh residents, including those who died out of Wales. This dataset is used to identify date of death for the Administrative table and for a residency end date for the Residency supplementary cohort
**Annual District Death Extract**	ADDE	Office for National Statistics register of all births in Wales. These data are obtained from the Office for National Statistics and are derived from information collected during civil registration, which is a legal requirement. This dataset is used to identify births for the Birth cohort. It does not contain maternal linkage
**Congenital Anomaly Register and Information Service**	CARS	Information about any foetus or baby who has or is suspected of having a congenital anomaly and whose mother is normally resident in Wales at time of birth. This dataset is used as a flag in the Administrative table
**Critical Care Dataset**	CCDS	Contains critical-care-specific fields, giving greater granularity for patients under critical care. This dataset is used as a flag in the Administrative table
**Emergency Department Dataset**	EDDS	Attendance and clinical information for all Accident and Emergency attendances. This dataset is used as a flag in the Administrative table
**Education Wales**	EDUW	Schools and pupil data for Wales that cover state-funded learning centres. This dataset is used to create the Education outcomes and Educational well-being tables. It also used to derive the special educational/additional learning needs (SEN/ALN) and free school meals (FSM) flags in the Administrative table
**Looked After Children Wales**	LACW	Information about Looked After Children Wales. Includes demographic and episode-level information. This dataset is used as a flag in the Administrative table
**Maternity Indicators Dataset**	MIDS	Captures data relating to the woman at initial assessment and to mother and baby (or babies) for all births. This relates to initial assessment and birth activity undertaken in Wales only. This dataset is used to identify births for the Birth cohort and mothers for the Mother cohort
**National Community Child Health**	NCCH	The Child Health System in Wales; includes birth registration and monitoring of child health examinations and immunizations. This dataset is used to identify births for the Birth cohort and mothers for the Mother cohort
**Outpatient Database for Wales**	OPDW	Attendance information for all hospital outpatient appointments. This dataset is used as a flag in the Administrative table
**Patient Episode Dataset for Wales**	PEDW	Contains all inpatient and day-case activity undertaken in NHS Wales plus data on Welsh residents treated in English Trusts. This dataset is used as a flag in the Administrative table
**Welsh Demographic Service Dataset**	WDSD	Register of all individuals registered with a Welsh GP that includes individuals’ anonymized address and practice history. This dataset is used to identify all children who lived in Wales while aged <18 years for the Child cohort, to confirm that mothers in the Mother cohort have lived in Wales. It is also used to identify residencies and co-residents for the Residency supplementary cohort and a number of demographic variables (e.g. WIMD). Finally, it is used as a flag in the Administrative table
**Welsh Longitudinal General Practice Dataset**	WLGP	Register of all individuals registered with a Welsh GP that includes individuals’ anonymized address and practice history. This dataset is used as a flag in the Administrative table
**Welsh Results Reports Service**	WRRS	Allows healthcare professionals across Wales to access laboratory results for pathology requests and any other associated results across all health boards in Wales, wherever they had their test taken. This dataset is used as a flag in the Administrative table

Some fixed demographic information is stored in the main three sub-cohorts, but the remainder is held in a series of derived supplementary tables that capture domains not directly contained within the core sub-cohorts, reducing the need for researchers to prepare them independently. These include an Administrative table containing fixed, harmonized demographic measures such as ethnic group [24], week of birth, date of death, and sex, alongside flags for data quality and availability, and information on cohort entry and exit. These data-availability flags indicate which other SAIL data sources these individuals appear in, covering a range of administrative, health, education, and social-care aspects. The education resources consist of two tables: one covering educational outcomes from Key Stage assessments (including the Foundation Phase) and another capturing indicators of educational well-being, such as attendance, school mobility, special educational needs (SEN) (up to 2021) and additional learning needs (ALN) (2021 onwards), and staffing ratios. The Looked After Children table records episodes of local authority care, supporting longitudinal analyses of social-care experiences. Finally, a supplementary residency cohort provides residential histories, including RALFs, residency dates, and co-resident ALFs, enabling the identification of household members.

Area-level measures are captured from residential linkage (RALFs) and include the Welsh Index of Multiple Deprivation (WIMD)—the official deprivation index for Wales, which uses an aggregate score to rank all 1909 Lower-layer Super Output Areas (LSOAs) from least (1) to most deprived (1909), aggregated into deciles [25]—and the Townsend Deprivation Index, which is another widely used area-based measure derived from UK census data [26].

CWTCH also includes a rich set of individual-level or household-linked proxy indicators of deprivation. Eligibility for free school meals (FSM) is a widely used proxy for low household income [27], although researchers should consider policy changes over time that may have affected its interpretation and comparability [28]. Educational indicators such as SEN or ALN status, individual pupil-level data including education attainment scores (Key Stages 1–4 and Foundation Phase), attendance rates (authorized and unauthorized absences), school mobility, and exclusion records provide insight into structural disadvantage and educational engagement. Residential mobility can be drawn from RALFs to support the analysis of housing stability and to derive measures such as household composition from co-residency patterns. Social-care data, including child-protection registrations, looked-after children records, and referrals to children’s services, are also available where linkage permissions allow.

A key strength of CWTCH is its capacity, where permissions allow, to link to census data. This provides access to more granular and robust measures of household socio-economic circumstances than are typically available in routine administrative datasets. These include household employment status (e.g. part-time, full-time, unemployed), occupational class (National Statistics Socio-Economic Classification), highest educational qualification (e.g. no qualifications through to degree level), and housing tenure (e.g. owned, privately rented, social housing). Additional variables capturing migration status, ethnic groups, language spoken at home, types of disability, or limiting long-term illness support the analysis of other axes of inequality associated with heightened exposure to disadvantage, as well as intersectional analyses.

Finally, the maternal linkage structure in CWTCH captures mothers’ socio-economic conditions before, during, and after pregnancy, including antenatal-care records, maternal diagnoses (e.g. gestational diabetes, hypertensive disorders of pregnancy), smoking status during pregnancy, and parity. This enables the intergenerational analysis of how these factors shape the health, well-being, and socio-economic circumstances of their children and, as those children move into adulthood and become parents, their grandchildren.

## Data resource use

CWTCH is constructed within the SAIL Databank TRE, facilitating linkage with administrative, health, education, and care data sources to support the analysis of structural and compositional inequalities throughout the life course and across generations. SAIL enables the investigation of a wide range of child exposures and outcomes, including perinatal and early-life indicators (e.g. perinatal factors, birth outcomes, immunizations), physical and mental health diagnoses, healthcare utilization, educational attainment and attendance, SEN, and children’s social-care involvement.

**Figure 2:**
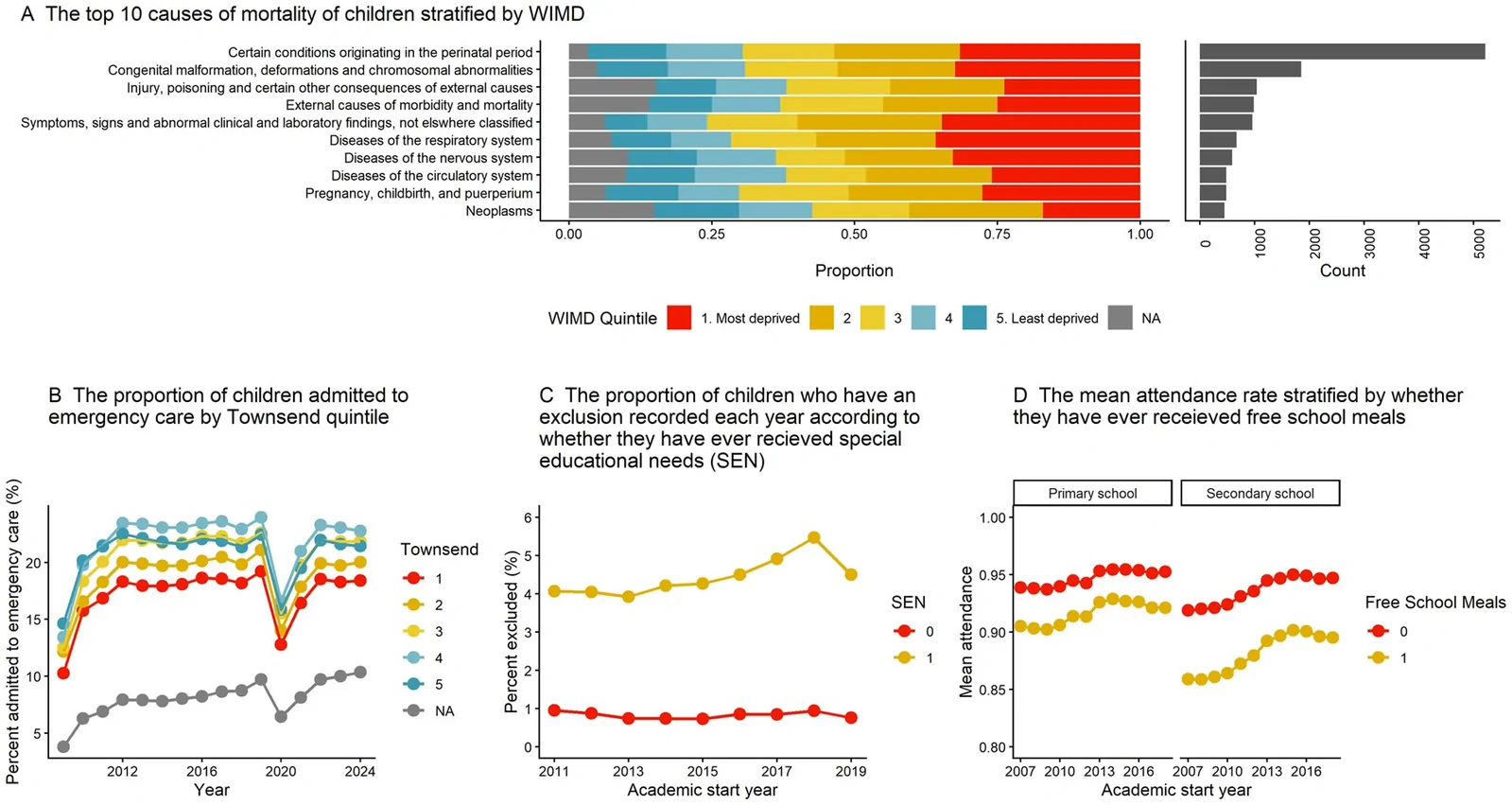
How data from the Birth sub-cohort could be used to investigate inequalities in child health and education. We present four examples of how the Birth sub-cohort could be used to investigate inequalities: (a) Mortality and the top 10 causes of death stratified by the Welsh Index of Multiple Deprivation (WIMD) quintile. (b) The proportion of children who are admitted to emergency care each year stratified by the Townsend quintile. (c) The proportion of children who have an exclusion record each academic year stratified by whether they have ever been recorded as having special educational needs (SEN). (d) The mean attendance of students in primary and secondary schools each year stratified by whether they have ever received free school meals.

To illustrate the analytical potential of CWTCH, we present four descriptive examples spanning health and education domains and multiple axes of inequality (Fig. 2). These examples were selected to demonstrate the breadth of linked data available and the types of analyses that the resource can support, rather than to address specific research questions.

The selected examples also reflect current research and policy priorities in Wales (Fig. 2). Persistent school absence among FSM-eligible pupils illustrates how CWTCH can support the evaluation of policies aimed at improving school attendance and reducing educational inequalities associated with child poverty. Emergency admissions by Townsend deprivation quintile demonstrate the potential to investigate socio-economic inequalities in healthcare utilization and evaluate policies focused on prevention and reducing avoidable hospital admissions. Fixed-term exclusions by SEN or ALN status highlight opportunities to assess the impact of ALN reforms and wider inclusion policies on educational outcomes. Child mortality by WIMD demonstrates the capacity of CWTCH to monitor inequalities in child health and support research that informs strategies to reduce avoidable differences in outcomes across the life course.

Collectively, these examples demonstrate the ability of CWTCH to support longitudinal, cross-sector analyses by using multiple, complementary measures of inequality. By integrating routinely collected health, education, and social data, the resource provides a platform for evaluating policy interventions, monitoring population inequalities, and investigating life-course pathways that influence child health and well-being in Wales.

Beyond these illustrative applications, CWTCH is already being used to address a range of active research questions. Table 4 summarizes current projects utilizing the resource, including their research questions, objectives, and planned or ongoing analyses.

**Table 4:** Overview of ongoing and planned research projects using the CWTCH data resource, including research questions, study objectives, methodological approach, and current stage of development.

Research question	Study objectives	Short description of methodology	Why CWTCH is important
**How do age, sex, and socio-economic circumstances influence mental health outcomes in children and adolescents?**	To estimate the association between sex, age, household deprivation, and rates of mental health incidence in children and adolescents in Wales	Age-specific incidence rates and incidence rate ratios were modelled by using quasi-Poisson regression with a log-time offset, comparing sexes and deprivation quintiles against the least deprived. Eight conditions were analysed individually, plus a combined ‘any mental health diagnosis’ outcome. Individuals were followed from birth until their eighteenth birthday	CWTCH provides a standardized cohort spine, allowing selection of the Birth sub-cohort by using the recommended inclusion criteria to generate the analysis cohort
**How are maternal household socio-economic circumstances associated with the timing of first observed Child Protection Register (CPR) registration across childhood?**	To estimate the association between household deprivation and age-specific hazard rate of first observed CPR registration and to examine how this association changes with adjustment for demographic, perinatal, and maternal characteristics. Secondary objectives were to assess the population relevance of deprivation categories and to determine whether time-varying associations qualified the interpretation of the Cox-model estimates	This population-based linked birth cohort study followed 106 365 children born in Wales during 2010–12 from birth until first observed CPR registration, death, or administrative study end, using child age as the survival timescale. Household deprivation was derived from linked maternal 2011 Census records and analysed by using staged Cox regression, supplemented by attributable-fraction estimation and variable- and category-specific Schoenfeld-residual diagnostics	CWTCH constitutes the project’s core longitudinal data spine by providing secure mother–child linkage and encrypted identifiers across birth and maternity, demographic and GP registration, death registration, residence, and household-composition records. This dataset enables consistent linkage of maternal census socio-economic circumstances and child social-care outcomes, thereby supporting temporally ordered household-level analyses across otherwise fragmented administrative sources
**How do mental health diagnoses intersect with social-care involvement across the life course?**	To describe the population burden of first recorded mental health diagnoses by level of social-care involvement, including age- and sex-specific incidence rates and cumulative incidence To examine the timing and temporal ordering of first recorded mental health diagnoses in relation to social-care involvement To investigate socio-economic and ethnic inequalities in recorded mental health diagnoses by level of social-care involvement To explore the relationship between recorded adverse childhood experiences (ACEs), social-care involvement, and first recorded mental health diagnosis	Descriptive statistics and data visualization will be used to characterize patterns of mental health diagnoses and social-care involvement, with regression-based approaches used to examine associations between social-care involvement, recorded adversity, and mental health outcomes	CWTCH provides rich, linked data on children in Wales, their mothers, and household members, including demographic variables, social-care records, and childhood adversities recorded in electronic health records, paving the way for more causally informed analyses of the mental health effects of care experience
**How does exposure to adverse local-authority-level macroeconomic conditions during pregnancy influence the timing of mental health outcomes from birth to late adolescence?**	To estimate the association between prenatal exposure to local-authority-level claimant rates and the age-specific hazard of first occurrence of a range of mental health outcomes, using linked sibling data to account for shared familial factors	Continuous-time hazard models will be used with linked sibling data to examine a range of mental health outcomes, including depression and anxiety, self-harm, alcohol- and substance-use disorders, conduct disorders, and eating disorders	CWTCH provides rich, linked data on the full population of children in Wales born from 2000 onwards and their mothers, allowing siblings to be identified through maternal links and linkage to mental health records for a range of outcomes through late adolescence. Information on maternal residence allows exposure to local-authority-level monthly claimant rates during pregnancy to be assigned to each child
**Do school experiences of children known to social care influence their educational progress?**	To understand how school experiences can act as risk or protective factors for key educational progress measures, including attainment, attendance, and behaviour, by linking administrative data to survey data	Administrative data (CWTCH) will be used as the baseline cohort, linked to other sources, including social care, education, health, and wider survey data	CWTCH will provide a clean spine for data scientists to develop a linked cohort for this analysis

## Strengths and weaknesses

This paper summarizes the development of CWTCH—an RRDA constructed using the SAIL Databank. CWTCH represents the first population-scale cohort within SAIL explicitly designed to investigate child health, social, and educational inequalities in Wales. The inclusion of multiple domains of inequality enables multidimensional analyses to examine pathways to inequality, co-occurring health, social and educational outcomes, and the intergenerational transmission of poor health associated with social disadvantage.

A key strength of CWTCH is its population-level scope, with minimal inclusion criteria, enabling broad and largely representative coverage of children and mothers in Wales. This reduces selection bias relative to traditional cohort studies and supports the analyses of rare outcomes and population subgroups. Unlike previous Welsh cohorts, the absence of an upper age limit enables the analysis of outcomes in adolescence, such as further education, employment, and pregnancy [20]. The cohort links children to mothers and households, facilitating household-level and longitudinal analyses, such as the association of outcomes with policy changes or the intergenerational transfer of inequalities. The RRDA aspect is particularly valuable, with preloaded derived tables streamlining inequalities research by providing standardized indicators ready for analysis.

The breadth of routinely collected data supports the analysis of policy-relevant outcomes, including mortality, healthcare utilization, educational attainment, and social-care involvement, offering a comprehensive view of population health and well-being. GP registration minimizes attrition (16.4% of children, 12.4% of mothers lost to follow-up) and selection bias. CWTCH currently contains 970 880 children and 475 010 mothers, with its dynamic structure accommodating ongoing changes through births, migration, and deaths.

However, several limitations should be considered. Paternal linkage is currently unavailable, limiting the direct analysis of paternal influences; however, household-level information derived from residential linkage may partially mitigate this. As with all routine data, issues such as missing data or miscoding can arise, though population-level scale and multiple data sources help to mitigate these challenges. Future linkage to cohort or panel data could address the limitations inherent in routine sources.

Comprehensive documentation and openly available code on GitHub promote transparency and reusability, though familiarity with R, SQL, and the SAIL TRE is recommended for effective use.

## Data resource access

CWTCH represents an open-source, flexible, and scalable resource for investigating maternal and child health, educational outcomes, and social inequalities in Wales. The cohort can be refreshed as new data become available within the SAIL Databank, subject to project-specific approvals and funding. Researchers can replicate or adapt the cohort by using openly shared code and documentation available via https://github.com/SwanseaUniversityDataScience/RRDA_CWTCH, allowing them to tailor the resource to their research objectives.

Data are available in the SAIL Databank at Swansea University, but are not publicly accessible. All proposals undergo independent review by an Information Governance Review Panel (IGRP) and approved access is granted through a privacy-protecting TRE via the SAIL Gateway. The application process is available at https://saildatabank.com/data/apply-to-work-with-the-data/.

## Ethics approval

Approval for the use of anonymized data in this study, provisioned within the SAIL Databank, was granted by an independent IGRP under project 1409. The IGRP has a membership comprising senior representatives from the British Medical Association (BMA), the National Research Ethics Service (NRES), Public Health Wales, and Digital Health and Care Wales (DHCW). The usage of additional data was granted by each respective data owner. The SAIL Databank is compliant with General Data Protection Regulations (GDPR) and the UK Data Protection Act.

## Supplementary Material

dyag180_Supplementary_Data
