# Supplementary material for: Data Resource Profile: Children in Wales: The Cohort for Health and Wellbeing Inequality (CWTCH)
Source: Int J Epidemiol. 2026 Sep 10;55(5):dyag180. doi: 10.1093/ije/dyag180 (PMC13561516; doi:10.1093/ije/dyag180)
Supplement: Supplementary file 1 [file dyag180_supplementary_data.docx]

Supplementary material for CWTCH cohort

Contents

[S1 Supplementary tables:	1](#_Toc235619045)

[S2 Measures of socioeconomic inequality included in CWTCH	2](#_Toc235619046)

[Area-level measures	2](#_Toc235619047)

[Individual- and household-level proxies	3](#_Toc235619048)

[Intergenerational dimensions	3](#_Toc235619049)

[References	4](#_Toc235619050)

# S1 Supplementary tables:

The **Administrative table** includes harmonised demographic variables such as ethnicity, week of birth, date of death, and sex. Where available, variables like ethnic groups are derived from multiple sources to improve completeness. This table also includes several flags associated with data quality and data availability in various data sources. Where possible, variables like ethnicity are compiled from multiple sources to improve completeness

The **Education tables** consist of two separate resources: one capturing educational outcomes from Key Stage assessments (KS1–KS4), including the Foundation Phase which replaced KS1, and another covering indicators of educational wellbeing such as attendance, school mobility, special educational needs (SEN) and Additional Learning Needs (ALN) which replaced the SEN system, and staffing ratios.

Linkage with education data allows for analysis of school attendance and attainment. However, educational assessment data are not fully available throughout the cohort coverage period, and researchers should consider these data gaps when designing their research questions (Figure 1). The educational system for the early years changed in 2011, whereby the Foundation Phase replaced Key Stage 1. Both measures have a threshold identifying where children meet the “expected level” which can be used in analyses; however, they are not directly comparable.

Figure 1 Key Stage (KS) 1 is available from 2006 to 2010, with the replacement Foundational Phase assessment available from the following year (2011) until 2018, KS2 is available from 2010 to 2018, and KS4 from 2015 until 2018

The **Looked After Children** (LAC) table records episodes of care involving children placed in local authority care, enabling longitudinal analysis of social care experiences.

A separate **Residency supplementary cohort** provides details on residential histories. This includes all RALFs associated with a child, alongside start and end dates of residency and co-resident ALFs. This table facilitates the identification of household members, including siblings and caregivers, by indicating their co-location at the same address.

# S2 Measures of socioeconomic inequality included in CWTCH

## Area-level measures

The primary measure of area-based socioeconomic inequality within CWTCH is the Welsh Index of Multiple Deprivation (WIMD). WIMD is the official deprivation index for Wales and ranks all 1,909 Lower-layer Super Output Areas (LSOAs) within the 2011 version of the LSOA, each representing approximately 1,600 residents, from least (1) to most deprived (1909)[1]. It is a composite index calculated from eight weighted domains (Table 1). The measure is re-released approximately every 5 years, and SAIL holds records for the 2014 and 2019 versions. These WIMD scores are typically aggregated into quintiles for analysis.

Table 1 The WIMD weighted domains as presented by Welsh Government [2]

|  | WIMD 2014 domain weight | WIMD 2019 domain weight |
| --- | --- | --- |
| Income | 23.5% | 22% |
| Employment | 23.5% | 22% |
| Health | 14% | 15% |
| Education | 14% | 14% |
| Access to services | 10% | 10% |
| Housing | 5% | 7% |
| Community safety | 5% | 5% |
| Physical environment | 5% | 5% |

Researchers can also access domain-specific scores to explore specific dimensions of deprivation. For example, the income domain is frequently used as a proxy for household-level poverty, while the education domain may be used to explore area-level educational disadvantage. However, caution is advised when incorporating certain domains into outcome analyses. For instance, the health domain includes indicators such as cancer incidence, mortality, and limiting long-term illness, which may confound many health outcomes of interest. Researchers are encouraged to either exclude the health domain from composite measures or to use individual domains as covariates, depending on their analytical question[3].

The cohort also includes the Townsend Deprivation Index, another widely used area-based measure derived from UK census data[4]. Unlike WIMD, Townsend does not include health-related metrics and is based on four equally weighted variables: unemployment, lack of car ownership, non-home ownership and overcrowded housing. Townsend scores are linked to LSOAs and may serve as a useful alternative to WIMD, particularly for researchers seeking to avoid health-related circularity in deprivation measures or compare with other UK nations.

## **Individual- and household-level proxies**

CWTCH also includes several individual-level or household-linked indicators of deprivation. Free School Meal (FSM) eligibility is a widely used proxy for low household income[5]. However, users should familiarise themselves with policy changes affecting FSM eligibility, as these may impact the consistency and utility of FSM in this context[6]. SEN/ALN status, school mobility, and attendance rates can reflect broader disadvantage affecting educational stability and access. Residential mobility can be drawn from RALFs to support analysis of housing stability and to derive measures such as household composition from co-residency patterns. Given the limited availability of robust household-level socioeconomic measures in other UK data sources and the declining utility of FSM eligibility as a proxy, linkage to census data represents a key strength of CWTCH. Where permitted, researchers may access a richer set of household-level socioeconomic exposures, including employment status, occupational class, highest educational qualification, and housing tenure. Variables capturing migration status and disability supports analysis of other axes of inequality associated with heightened exposure to disadvantage, as well as intersectional analyses.

## **Intergenerational dimensions**

The maternal linkage structure in CWTCH captures mothers’ socioeconomic conditions before and during pregnancy, enabling intergenerational analysis of how these factors shape the health, wellbeing, and socioeconomic circumstances of their children – and, as those children move into adulthood and become parents, their grandchildren.

# References

1.	Welsh Index of Multiple Deprivation (WIMD): identifying groups of small areas based on deprivation indicators [HTML] | GOV.WALES. 2 Apr. 2025. https://www.gov.wales/welsh-index-multiple-deprivation-wimd-identifying-groups-small-areas-based-deprivation-indicators-html (24 Sept. 2025, date last accessed).

2.	Welsh Government. *Welsh Index of Multiple Deprivation (WIMD) 2019 Guidance*. Welsh Index of Multiple Deprivation. n.d. https://www.gov.wales/welsh-index-multiple-deprivation (24 Sept. 2025, date last accessed).

3.	Mohammed S, Bailey GA, Farr IW *et al.* Using the Welsh Index of Multiple Deprivation in research: estimating the effect of excluding domains on a routine health data study. *BMC Public Health* 2025;**25**(1):1178. https://doi.org/10.1186/s12889-025-22369-0.

4.	Gartner A. *Briefing Paper on LSOA Townsend Deprivation Scores Calculated from Unadjusted Census Data*. n.d.

5.	Taylor C. The Reliability of Free School Meal Eligibility as a Measure of Socio-Economic Disadvantage: Evidence from the Millennium Cohort Study in Wales. *British Journal of Educational Studies* 2018;**66**(1):29–51. https://doi.org/10.1080/00071005.2017.1330464.

6.	Universal Primary Free School Meals (UPFSM) | GOV.WALES. 4 Sept. 2024. https://www.gov.wales/universal-primary-free-school-meals-upfsm (10 Oct. 2025, date last accessed).
